# Evaluating use of disease-modifying therapies for multiple sclerosis using claims data

**DOI:** 10.3389/frhs.2026.1714922

**Published:** 2026-03-10

**Authors:** Xiang Wang, Fei-li Zhao, David Newby, Shuchuen Li

**Affiliations:** 1 School of Biomedical Sciences and Pharmacy, College of Health, Medicine and Wellbeing, The University of Newcastle, Callaghan, NSW, Australia; 2 Beijing Health Economics Association, Beijing, China

**Keywords:** claims data, disease-modifying therapies (DMTs), drug utilisation, multiple sclerosis, postmarket monitoring, real world data (RWD)

## Abstract

**Objective:**

The aim of this work is to evaluate trends in the utilisation, cost, and switching patterns of disease-modifying treatments (DMTs) for relapsing-remitting multiple sclerosis (RRMS) under the pharmaceutical benefits scheme (PBS) in Australia from 2010 to 2021.

**Methods:**

A retrospective analysis was conducted using PBS claims data for 14 listed DMTs. Key outcomes included treatment uptake, switching behaviour, patient persistence, per-patient dosage, and direct drug costs.

**Results:**

The cohort comprised 2,315 RRMS patients initiating DMTs between 2010 and 2021 (73% women; mean age 41.9 years). Over the study period, the number of patients receiving DMTs increased 5-fold, while total PBS expenditure on DMTs rose 4.5-fold (from AUD 109.4M to AUD 492.9M), representing a compound annual growth rate of 13.6%—significantly outpacing overall PBS expenditure growth. High-efficacy (HE) DMTs progressively replaced low-efficacy (LE) therapies for both treatment initiation and switching. Median treatment persistence was higher for HE DMTs (25.5 months) compared to LE DMTs (20.8 months). HE DMTs generally incurred higher per-patient costs; however, elevated annual dosages of the LE drug interferon Beta-1a resulted in higher costs than some HE therapies.

**Conclusion:**

Rising PBS expenditure on DMTs was primarily driven by increasing patient numbers, shifts from low- to high-efficacy therapies, and longer treatment persistence. Analysis of individual DMT utilisation identified higher-than-expected use of interferon beta-1a relative to defined daily doses benchmarks, contributing to increased treatment costs. Overall, the findings demonstrate the value of claims data for postmarket monitoring of medicine utilisation and expenditure, and for informing policy review aimed at sustainable resource allocation.

## 1 Introduction

Multiple sclerosis (MS) is a chronic neurological disorder that can result in significant physical and cognitive impairment. There is currently no known cure. Relapsing-remitting multiple sclerosis (RRMS) is the predominant form of MS, characterised by periods of relapse followed by partial or full recovery (1). In Australia, the prevalence of MS has risen considerably in recent years, with over 33,000 patients living with it as of 2021. Notably, the most rapid growth in MS cases was observed between 2017 and 2021 (2), highlighting the increased demand for effective treatments. Although relapses cannot be entirely prevented, disease-modifying therapies (DMTs) represent the cornerstone of RRMS management, slowing disease advancement, reducing the frequency and severity of relapses, and improving patient outcomes (3).

Over the past decade, Australian RRMS patients have gained access to a broad range of DMTs through the Pharmaceutical Benefits Scheme (PBS), which provides subsidised medication for eligible residents. As a part of the universal healthcare arrangements, patients only need to pay minimal or no cost to access the treatments listed in the PBS schedule. To support the PBS in achieving its dual objectives of delivering optimal health outcomes while ensuring economic sustainability, the Pharmaceutical Benefits Advisory Committee (PBAC), as legally mandated, reviews and provides recommendation to the Minister of Health before medicines can be listed on the PBS (4).

Rational use of medicines and the effective allocation of health resources are essential to ensure optimum health outcomes and sustainability of the health system. This involves ensuring that medications are appropriate for clinical needs, provided in the correct doses, for adequate durations, and at the lowest possible cost to both patients and the health system (5). The PBAC plays a crucial role in promoting the rational use of medicines in Australia by recommending medicines that are considered cost-effective. It conducts health technology assessment (HTA) to compare the effectiveness, safety, and cost-effectiveness of new medicines against current alternatives, and makes evidence-based recommendations for inclusion in the PBS (6). Nevertheless, the HTA evaluation of the PBAC heavily relies on efficacy and safety evidence from controlled experimental settings and experience-based assumptions about economics, such as those related to the disease prevalence, volume, and uptake. Such evidence inherently carries uncertainties and limitations (7). In 2015 and 2022, the Drug Utilisation Sub-Committee (DUSC) of the Pharmaceutical Benefits Advisory Committee (PBAC) conducted postmarket reviews of the newly included DMTs and identified disparities between real-world usage patterns and initial estimations used in the evaluations (8, 9). These findings highlight the need for postmarket surveillance to validate assumptions, assess real-world drug usage and effectiveness, and ensure that investments achieve optimal outcomes for both patients and the healthcare system. Ongoing monitoring is essential to ensure the rational use of listed medicines in accordance with clinical guidelines and HTA recommendations throughout their continued use.

As noted, the rising prevalence of MS, coupled with the expanding availability of DMTs, has reshaped the treatment landscape for RRMS. Consequently, these developments have driven a significant increase in PBS expenditures on DMTs, raising critical questions about rationality of usage and the efficiency of health investment in this therapeutic area. To address these questions, it is necessary to examine the drivers behind the rising expenditure, including increased disease prevalence, uptake of DMTs, shifts in usage patterns towards higher-cost therapies, longer treatment durations, improved patient persistence, and therapy switching due to patient preferences. Understanding these factors provides critical insights for stakeholders—particularly payers—to identify inefficiencies and implement targeted actions to optimise healthcare delivery while ensuring the sustainable allocation of resources.

The PBS has accrued extensive data on the claims and dispensing of drugs (10). This wealth of information has been leveraged across a broad spectrum of research topics (11). Within the MS therapeutic area, there have been a few attempts to leverage PBS data to investigate the prevalence of MS (12, 13) and the usage pattern changes (14–16). While these studies documented changes in drug usage patterns—including growth in patient numbers, shifts in the DMT landscape, and cost trends—they provided limited evidence on the rationality of DMT utilisation. In addition, they offered few insights into optimising drug use or improving resource utilisation. These limitations can be attributed to the datasets, which often lacked detailed patient-level information, necessitating assumptions to estimate outcomes, such as patient numbers (12–15). Moreover, the absence of fit-for-purpose analytical methods and indicators underscores the need for more robust, comprehensive analyses to generate deeper insights into the rational use of DMTs.

The objective of this study was to explore the use of claims data to address gaps in postmarket monitoring of rational medicine use and health resource allocation in real-world settings. In this study, we investigated several key areas related to the utilisation of drugs and healthcare resources from a payer's perspective, including drug usage relative to defined daily doses (DDD) to identify over- or under-utilisation, patient prevalence trends versus changes in drug usage patterns, treatment persistence (length of time patients remain on therapy), and the relationship between usage patterns and healthcare costs.

## 2 Method

### 2.1 Data sources and processing

Three data sources were used in this study. For PBS expenditure data, information was searched and extracted from the PBS item reports (17). These provided the information on DMT expenditure between 2010 and 2021. The DMT expenditure was compared with the total PBS expenditure, which was available through PBS expenditure and prescription reports (18). DMT patient and usage information was obtained from a 10% sample of PBS dispensing data supplied by Service Australia. Dispensing of DMTs for RRMS was identified using PBS item codes corresponding to DMT listings specific to RRMS. Prescriptions for these DMTs are subject to defined clinical eligibility criteria and authority requirements under the PBS. Based on their efficacy, DMTs were categorised as low-efficacy (LE) DMTs (Glatiramer Acetate, Interferon-Beta, Teriflunomide, and Dimethyl Fumarate) and high-efficacy (HE) DMTs (Natalizumab, Fingolimod, Ozanimod, Siponimod, Alemtuzumab, Cladribine, Ocrelizumab, and Ofatumumab) (19, 20). Differences in efficacy and associated costs lead to different outcome for patients and varied costs for the health system.

Unique patient identifiers were used for the identification of treatment initiation and switching events, and to determine the number of patients included in each treatment cohort for the on-treatment analyses. Patients included in this research were those who initiated their first DMT treatment between 2010 and 2021. The same cohort and their dispensing records were used for all analyses of drug uptake, usage, switching, persistence, and direct drug cost. The follow-up period extended until 31 December 2021.

The dispensed dosages were calculated by multiplying the number of prescriptions dispensed, the quantity dispensed, and the strength of the drug as outlined for each product. Patient usage was calculated by dividing the total dispensed dosage of the year by the number of patients who received at least one prescription of the drug in that year.

Defined daily dose (DDD) benchmarks for DMTs were derived from the World Health Organization ATC/DDD Index (21) and used to calculate annual dispensed DDDs per patient to compare utilisation patterns.

For the direct cost of each DMT, including varied forms or strengths of the same drug, the dispensed price for maximum quantity (DPMQ) was applied, as available from PBS website for each drug and item code. The DPMQ represents the total cost of the PBS, which includes the direct medicine cost, dispensing fees, and mark-ups. Recognising that DPMQ prices may have changed during the study period due to pricing reviews and regulatory adjustments, the 2022 price was applied uniformly to all DMTs in this analysis.

### 2.2 Data analyses

While DMT and overall PBS expenditures were derived from national expenditure reports, all patient-level utilisation analyses were based on the raw 10% PBS sample, with results presented as sample-based estimates to describe utilisation trends. Data were analysed and presented using Microsoft Excel 2016. Kaplan–Meier analysis of drug persistent was conducted using the Python lifelines package (22). The discontinuation of treatment was defined as patient either switching to another DMT or ceasing treatment due to death or other reasons; the data were right-censored.

## 3 Results

### 3.1 DMTs included in the study

A total of 14 DMTs for RRMS were listed on the PBS during the research period (Table 1). Four drugs—Interferon Beta-1b, Interferon Beta-1a, Glatiramer Acetate, and Natalizumab—were available prior to 2010, while the remaining 10 were progressively added before 2021.

**Table 1:** Disease-modifying treatments on the PBS.

DMT	Efficacy	Administration route	Frequency	Available on PBS
Interferon beta-1b	Low	Injection	Fortnightly	November 1996
Interferon beta-1a	Low	Injection	Weekly	February 1999
Glatiramer acetate	Low	Injection	Daily	July 2004
Natalizumab	High	Infusion	Monthly	October 2009
Fingolimod	High	Oral	Daily	August 2011
Dimethyl fumarate	Low	Oral	Daily	November 2013
Teriflunomide	Low	Oral	Daily	November 2013
Peginterferon beta-1a	Low	Injection	Fortnightly	March 2015
Alemtuzumab	High	Infusion	Yearly	April 2015
Ocrelizumab	High	Infusion	6 monthly	February 2018
Cladribine	High	Oral	Yearly	January 2019
Siponimod	High	Oral	Daily	November 2020
Ozanimod	High	Oral	Daily	June 2021
Ofatumumab	High	Injection	Monthly	October 2021

### 3.2 Patient cohort

Between 2010 and 2021, 2,315 patients had received their first DMT treatment and were included in the study (Table 2). Patient counts reflect individuals observed in the PBS data who initiated and received DMTs during the study period.

**Table 2:** Patient demographics.

Group	Number of patients	Percentage
Sex		
Female	1,694	73.17
Male	620	26.78
Unknown	1	0.04
Age^a^		
<18	21	0.95
18–34	691	29.85
35–45	693	29.94
45–54	518	22.38
55–64	274	11.84
65+	118	5.05
Mean age (SD)^a^	41.93 (12.77)	
Min. age	12	
Max. age	88	

^a^Age was calculated at the time the patient first initiated the treatment.

Among the cohort, 73% was women, and the average age at the time of initiation of DMT was 41.93 years. The majority of patients (57.9%) initiated treatment between the ages of 18 and 45 years, followed by patients aged between 45 and 54 years (22.38%). This patient profile aligns with the typical profile of MS patients.

### 3.3 The usage and expenditure trends of DMTs

**Figure 1:**
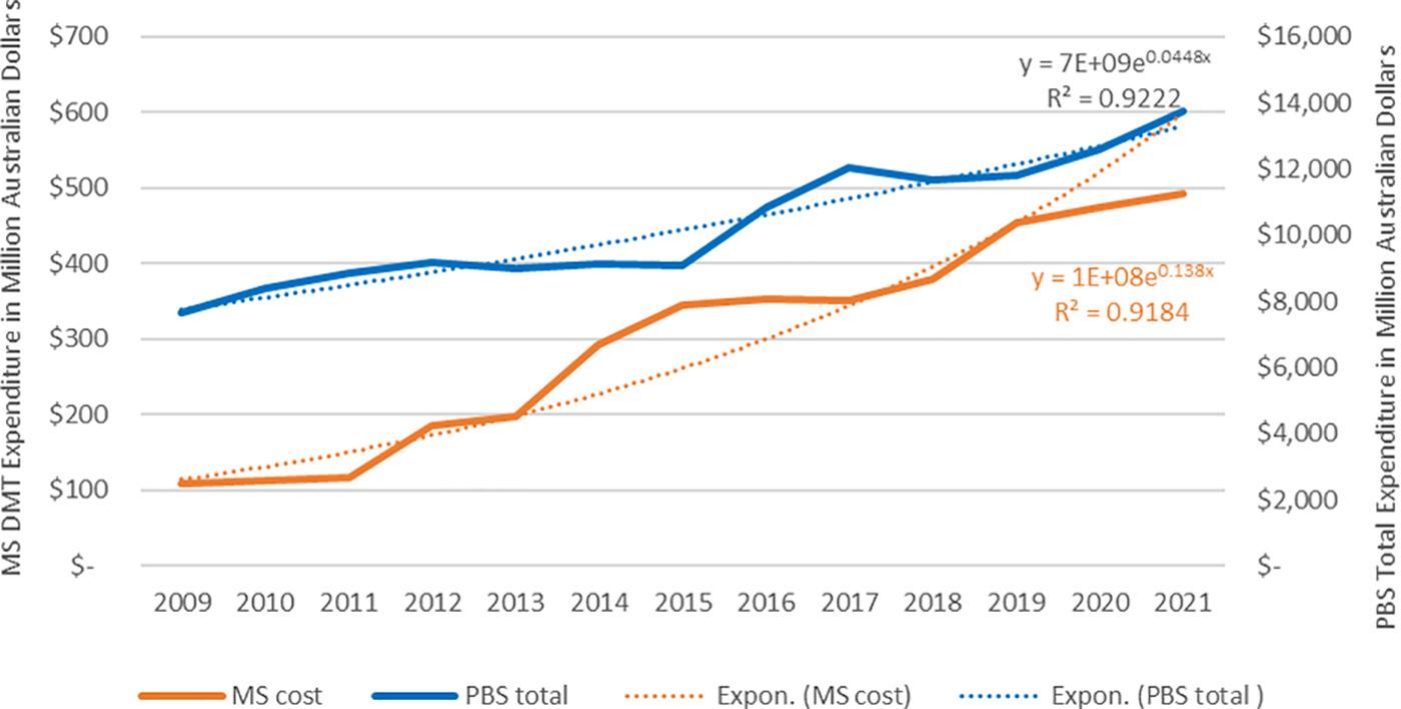
PBS expenditures on DMTs. The exponential regressions (dotted lines) represent the year-on-year change of MS and total expenditures.

**Figure 2:**
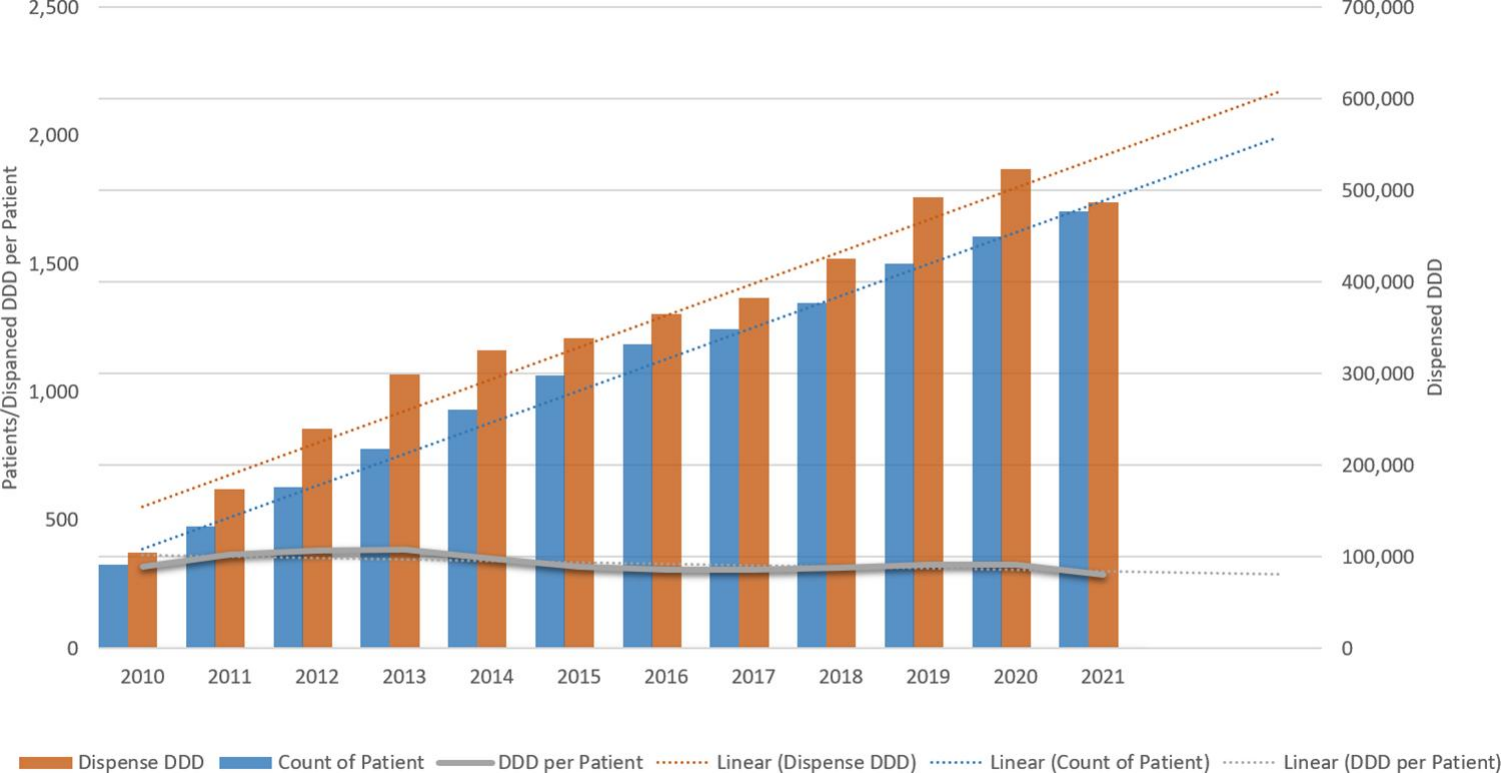
Trends in DMT uptake (patients on treatment) and number of DDDs dispensed. The linear regressions (dotted lines) present the trend of the total dispensed DDD, number of patients, and the average DDD per patient over the years.

Expenditure on DMTs increased 4.5-fold, from AUD $109.4M in 2010 to AUD $492.9M in 2021 (Figure 1, orange line and left *y*-axis). During the same period, the compound annual growth rate for DMT expenditure was 13.6% (Figure 1, dotted orange line), which was three times more than the 4.43% annual growth for total PBS expenditure (Figure 2, dotted blue line). This significant increase highlights the growing financial impact of DMTs on the PBS.

Concurrently, the number of patients receiving DMTs increased 5-fold, rising from 327 in 2010 to 1,705 in 2021 (Figure 2). This trend is in line with MS Australia reports indicating rising MS prevalence over time (2). The rising trend of total dispensed DDD aligned with the increase in patients (Figure 2, dotted lines of regressions). As a result, the average DDD dispensed per patient per year remained steady (Figure 2, dotted grey line).

### 3.4 The usage pattern of DMTs

**Figure 3:**
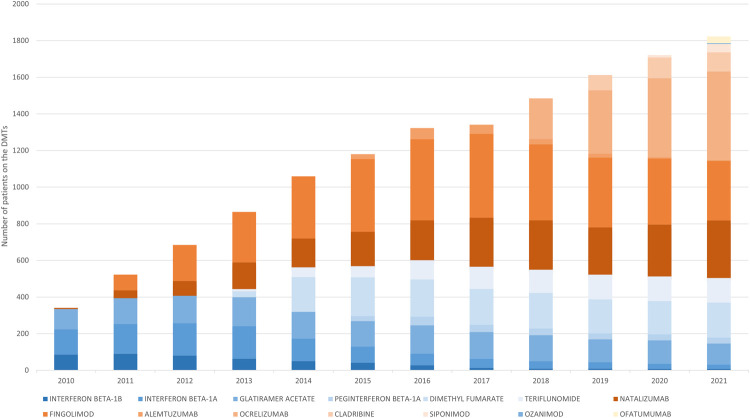
Annual trends in patients receiving DMTs. Number of patients who received at least one dispensation of the DMTs in each year.

During the study period, high-efficacy DMTs gradually replaced low-efficacy treatments. Since 2016, patients on high-efficacy DMTs have outnumbered those on low-efficacy DMTs, with those on low-efficacy DMTs continuing to decline (Figure 3).

**Figure 4:**
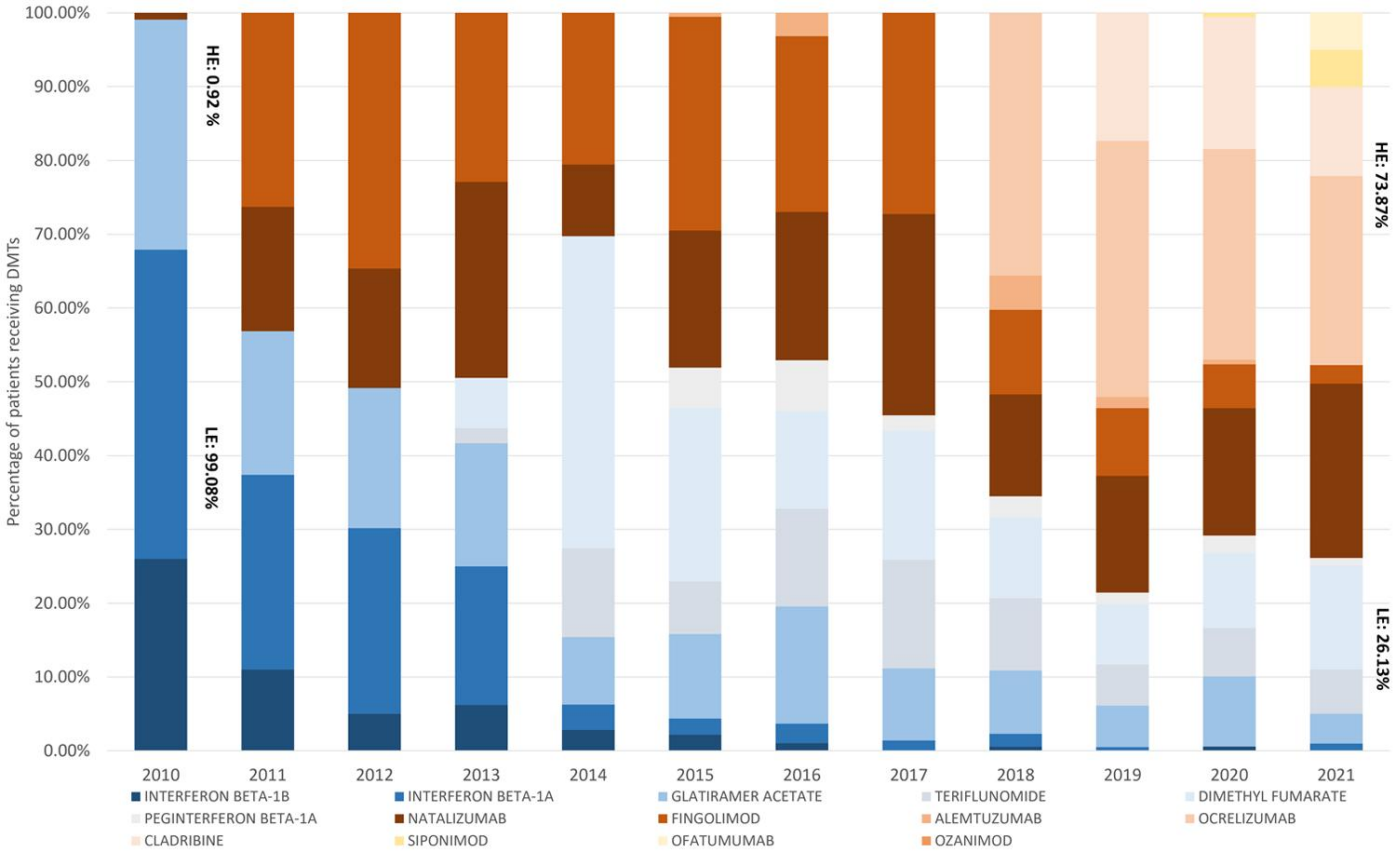
Initiator usage pattern. Proportion of patients receiving the varied DMTs in each year. LE, low-efficacy DMTs; HE, high-efficacy DMTs.

This shift from low-efficacy to high-efficacy DMTs was observed in both newly initiated patients (Figure 4) and those who started on LE DMTs and later switched to HE DMTs (Table 3).

**Table 3:** Switch pattern between efficacy groups.

To from	Low efficacy	High efficacy
Low efficacy	21.61% (360)	36.91% (615)
High efficacy	8.70% (145)	32.77% (546)

Number of switches *N* = 1,666, number of patients = 1,071.

LE treatments were substituted by HE therapies as the initial treatments for RRMS patients (Figure 4). Fingolimod and Natalizumab were the most used HE DMTs during the research period. Following the addition of new DMTs, the uptake of Natalizumab by new RRMS patients remained stable, while use of Fingolimod—the first oral DMT—was overtaken by other HE treatments, with Ocrelizumab emerging as the most used DMT by new RRMS patients.

Of the 2,315 patients studied, 1,071 (approximately 46%) used two or more DMTs (Table 3). A total of 1,666 treatment switches were recorded among those 1,071 patients during the study period. The majority of treatment switches involved HE DMTs: 36.91% of all switches were from LE to HE DMTs, while 32.77% were between different HE DMTs. This shift reflects a trend towards more patients and clinicians increasingly opting for high-efficacy therapies.

### 3.5 Annual dispensed doses of DMTs per patient

**Figure 5:**
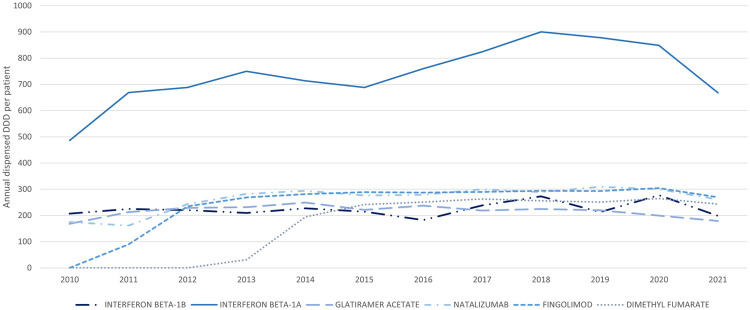
Annual dispensed DDD per patient for LE DMTs. Average annual number of DDDs per patient for low-efficacy DMTs.

**Figure 6:**
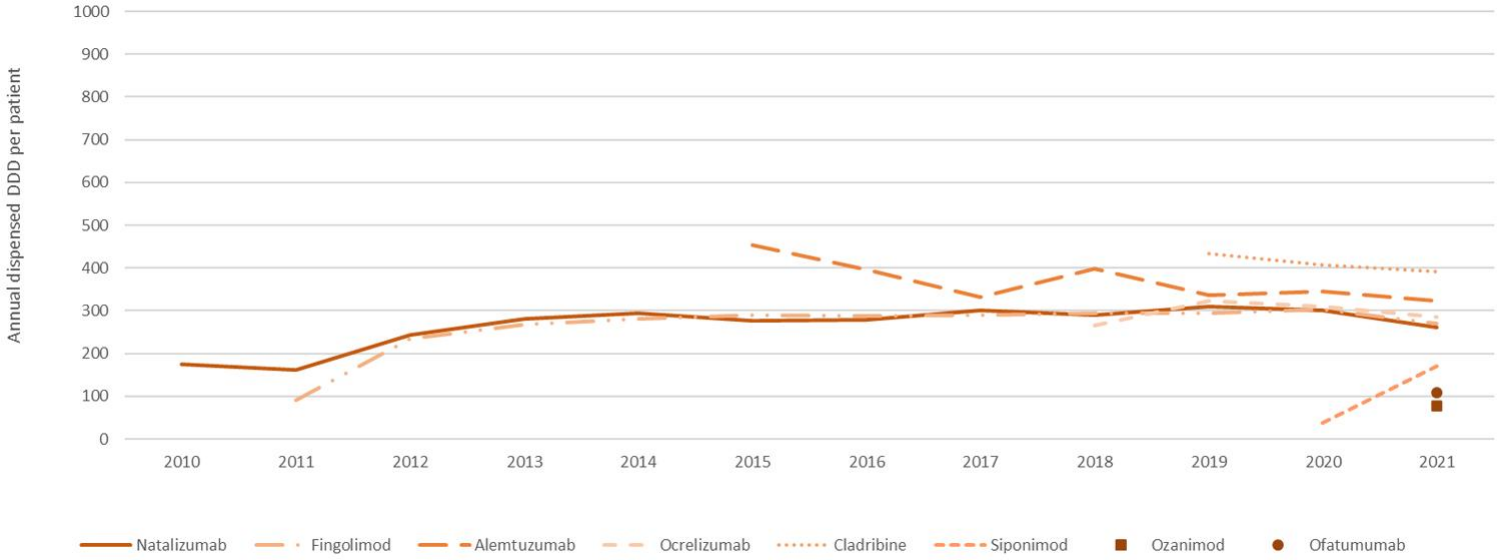
Annual dispensed DDD per patient for HE DMTs. Average annual number of DDDs per patient for high-efficacy DMTs.

Overall, the DDD per patient per year exhibited consistent patterns for each DMT, with variations observed among LE (Figure 5) and HE drugs (Figure 6). For new treatments listed in the PBS, it typically took about 1 year after initial listing for a drug to reach a quiescent period of DDD per-patient usage. Among all DMTs, interferon beta-1a was notably characterised by higher utilisation relative to DDD benchmark and variability in usage over time.

### 3.6 Direct drug cost

Based on the 2021 annual per-patient usage, the direct drug costs for different DMTs are presented in Table 4. The annual per-patient cost of high-efficacy DMTs was higher than that of low-efficacy drugs. Exceptions included dimethyl fumarate and interferon beta-1a, both of which had higher annual treatment costs than the HE DMTs. Interferon beta-1a, despite having a lower cost per DDD comparable to other DMTs, resulted in a higher overall cost due to its high annual DDD usage per patient. As of 1 April 2023, interferon beta-1a products (brand name REBIF and AVONEX) were delisted from the PBS (23), meaning that RRMS patients in Australia no longer have access to these subsidised treatments through the PBS.

**Table 4:** Direct annual drug cost per patient for DMTs.

DMT	Efficacy group	Average of DDD per patient (2021)	Cost per DDD	Annual cost per patient under the treatment
Teriflunomide	Low	251.48	$6.42	$1,614.76
Glatiramer acetate	Low	179.34	$27.03	$4,848.25
Interferon beta-1b	Low	198.75	$25.45	$5,058.45
Peginterferon beta-1a	Low	240.74	$23.80	$5,729.90
Dimethyl fumarate	Low	243.44	$37.63	$9,159.82
Interferon beta-1a	Low	667.91	$20.32	$13,570.09
Ofatumumab	High	107.03	$72.83	$7,794.42
Natalizumab	High	261.02	$31.24	$8,155.11
Fingolimod	High	269.35	$57.00	$15,351.63
Siponimod	High	170.31	$129.31	$22,023.36
Ocrelizumab	High	286.37	$91.33	$26,154.61
Alemtuzumab	High	323.08	$117.26	$37,885.05
Cladribine	High	390.76	$126.19	$49,308.58

### 3.7 Patient persistence on the DMTs

**Figure 7:**
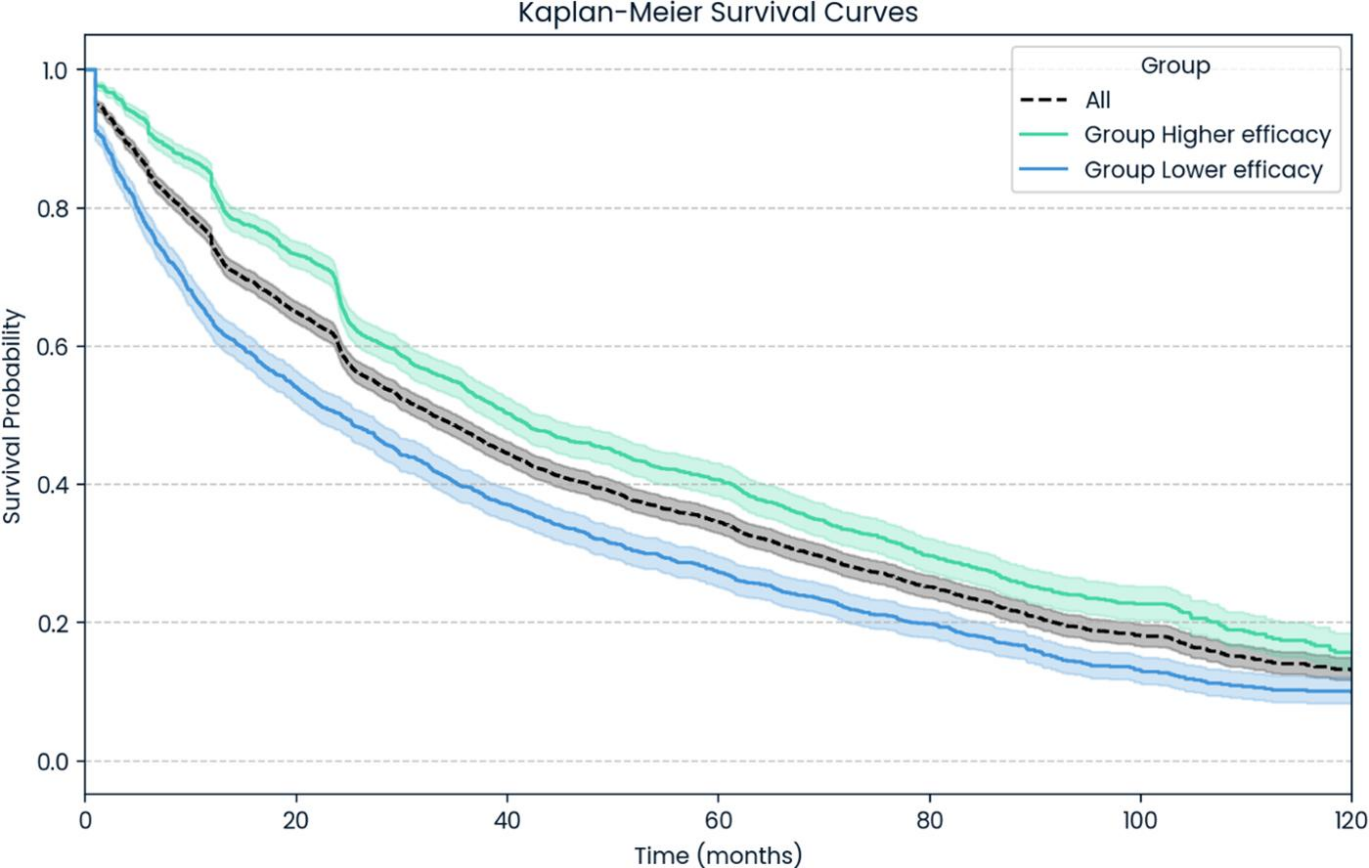
Persistence on the treatments. Statistics for all DMTs: median follow-up time (IQR): 24.64 months (10.13–51.00 months), mean duration on treatment (SD): 35.44 months (32.95 months). Statistics for group higher efficacy: median follow-up time (IQR): 25.49 months (12.45–49.93 months); mean duration on treatment (SD): 36.66 months (31.47 months). Statistics for group lower efficacy: median follow-up time (IQR): 20.80 months (6.34–52.44 months); mean duration on treatment (SD): 33.81 months (34.77 months).

Patients exhibited greater persistence on the high-efficacy DMTs compared with the low-efficacy treatments (36.66 months vs. 33.81 months). Kaplan–Meier analyses of treatment duration for LE and HE therapies are shown in Figure 7. It should be noted that LE DMTs were available on the market before the introduction of HE treatments.

### 3.8 DMT usage by administration routes and regimens

**Figure 8:**
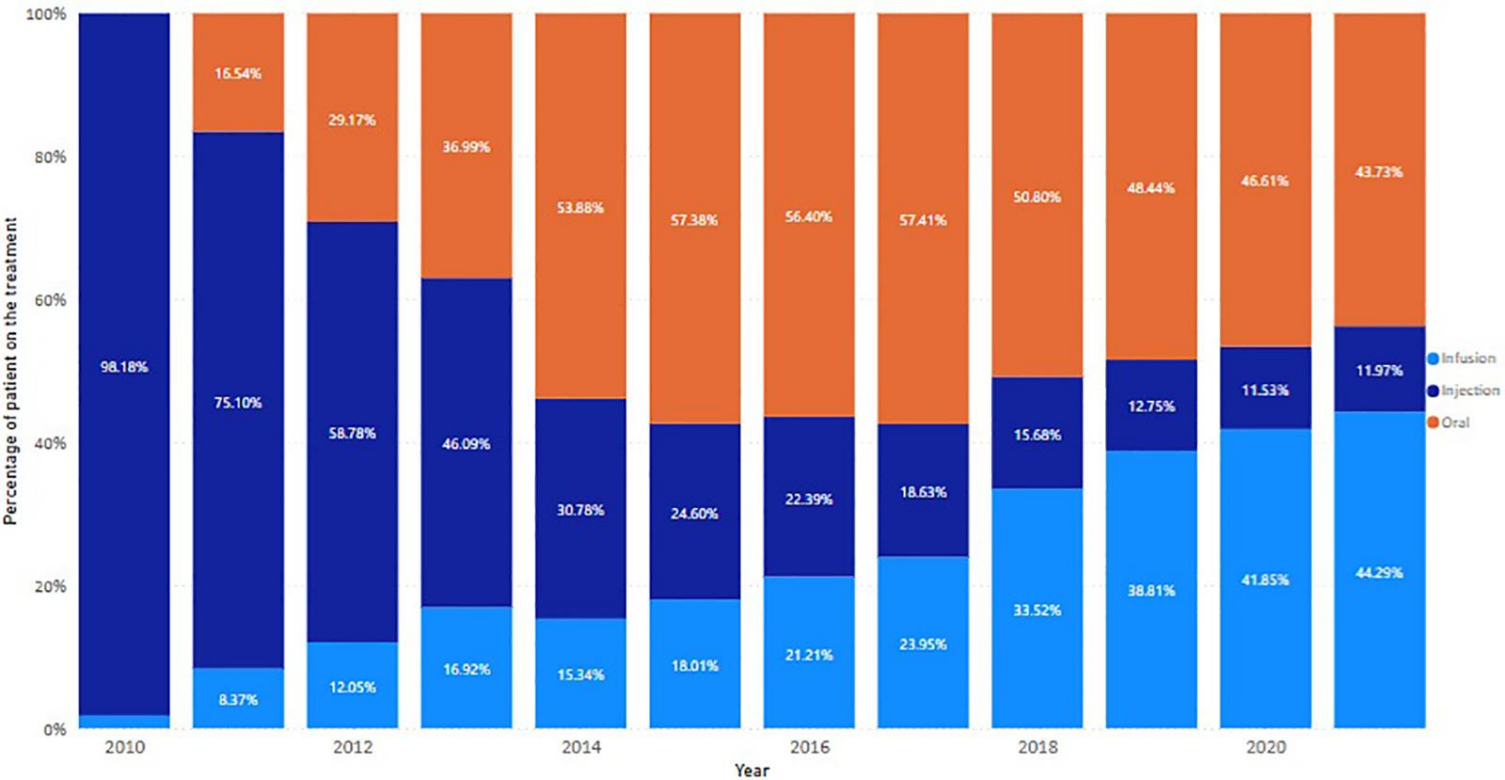
The trend of DMT usage by administration routes. Proportion of dispensed DMTs by administration route in each year.

**Figure 9:**
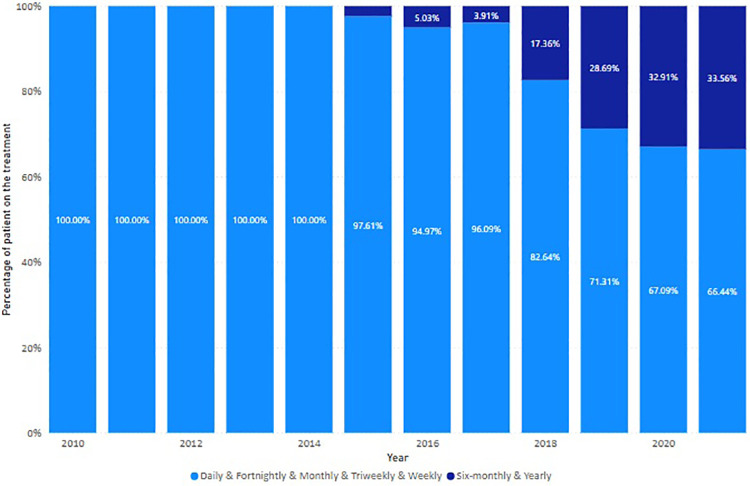
The trend of DMT usage by regimens. Proportion of dispensed DMTs by regimens in each year.

Since the launch of first oral DMT, Fingolimod, in 2011, the use of oral treatment increased, replacing conventional injection interventions (Figure 8). However, this trend changed after 2017 when the long-acting infusion treatment, Ocrelizumab, was listed on the PBS. Long-acting (6 monthly and yearly) treatments, including Alemtuzumab, Ocrelizumab, and Cladribine, progressively recaptured the market share for injectables (Figure 9).

## 4 Discussion

In Australia, healthcare spending on DMTs for RRMS increased significantly during the period between 2010 and 2021. The growth rate of DMT expenditure was three times higher than that of overall PBS drug spending during the same period. Given the rapid increase of costs in this therapeutic area, it is crucial for the stakeholders—particularly payers—to evaluate the rational use of DMTs and healthcare resources following the listing decisions. Such evaluation should include assessing alignment with clinical guidelines, appropriateness of dispensed doses, treatment duration, and the actual costs of these therapies. This retrospective real-world study investigated the patient and drug usage patterns of DMTs, providing valuable insights into the drivers of increased usage. In our study, three key factors contributing to the rise in expenditure were examined, namely, the number of patients receiving treatment, the use of different DMTs, and the costs associated with each treatment. Unlike previous studies (12, 13, 15, 16) that utilised PBS statistical data, a major strength of this study was the use of real-world PBS dispensing data, representing 10% of the population under PBS coverage. This dataset contained information at the individual patient level, allowing for more accurate assessment by eliminating the need for assumptions regarding patient numbers and per-patient drug usage. The use of this patient-level data aided comprehensive analyses of patient initiation, switching patterns, and treatment persistence for DMTs.

The number of patients observed receiving DMTs increased 5-fold over the study period. As demonstrated in our analysis, this was driven by both an increase in new patients initiating DMTs and high persistence rates among existing patients. Two main factors likely contributed to the initiation of DMTs for RRMS patients: (1) the growing prevalence of MS, mainly driven by improved diagnostic capabilities (2, 24), which enabled more patients to receive accurate and timely diagnoses; and (2) the recognition of DMT as the optimal treatment for RRMS (12, 25). Our analysis also found that the mean treatment duration was 36.66 months across all DMTs, with higher persistence among those on high-efficacy DMTs compared to low-efficacy options. This aligns with previous research showing that patients on DMTs exhibit high levels of adherence and persistence (26). In addition, our results indicated that despite the availability of new treatments and the increasing number of patients receiving the DMTs, the average annual per-patient dosage—measured as dispensed DDD per patient per year—remained stable for most DMTs. This suggests that the increase in patient numbers and strong treatment persistence were the primary drivers behind the overall rise in DMT usage. Previous research using the PBS statistics data reported overall increased disease prevalence and use of DMT among RRMS patients (12). Our approach advances prior work by using individual patient data to quantify and distinguish the underlying drivers of increased drug utilisation.

While the overall trend in DMT use aligned with the rising RRMS patient population, our study of the annual dispensed doses per patient offered insights into the use of each DMT. To our knowledge, this is the first Australian study to report real-world average use per patient. Notably, our study identified higher-than-expected utilisation of interferon beta-1a (non-pegylated interferon beta-1a) relative to DDD benchmarks among Australian patients. A similar pattern has been reported in Germany, where higher-than-average use of peginterferon beta-1a was observed (27). This finding signals consistent high dosage and potential financial implications associated with this medicine. It is worth noting that the PBS delisted two Interferon Beta-1a products for RRMS treatment in April 2023: Rebif® in December 2022 and Avonex® (23). Our findings of atypically high utilisation of interferon beta-1a provide supportive real-world evidence that is consistent with the PBS decision to delist this therapy. In addition, by 2021, only 24 patients—representing 1.32% of the patient population that year—were on this treatment. The low number of patients receiving this therapy indicates that any potential removal likely had a minimal impact on the overall patient population. Although our findings retrospectively concurred with the PBS decision, the insights into real-world usage and patient patterns offer compelling evidence in support of periodic routine reviews and potential delisting decisions, particularly for high-cost drugs and drugs prompted for atypical patient-level utilisation. This finding highlighted the effectiveness of our approach and measurement framework as a effective tool for identifying and informing the divergent use of medicine and health resources.

With more HE DMTs introduced to the PBS over the years, higher treatment costs were observed, as expected. In this study, we analysed the real-world per-patient drug cost based on actual DDD usage. The results indicated that annual per-patient drug costs were notably higher for HE DMTs compared to LE DMTs, with only a few exceptions for LE dimethyl fumarate and interferon beta-1a. The high annual cost of the latter was due to the exceptionally high dosage, as discussed previously. In addition, our findings also indicated greater uptake of HE DMTs. Patients who initiated treatment with an LE DMT and switched to an HE DMT followed what is known as an escalation treatment strategy. Conversely, patients who began treatment directly with an HE DMT followed an induction treatment strategy. In Australia, the induction approach is recommended by the Therapeutic Guidelines® for high-risk RRMS patients to achieve the target of NEDA (no evidence of disease activity) (28). Our study observed a rising trend in the uptake of HE DMTs, consistent with guideline recommendations. By 2021, patients were three times more likely to start with HE DMTs than with LE DMTs. Throughout the study period, when treatment switching occurred, the majority of patients transitioned to high-efficacy DMTs as the second- or third-line treatment choices. The rising cost of care for RRMS has been driven not only by the growing patient population but also by increased HE DMT usage, superior persistence on these therapies, and their higher costs. The use of HE DMTs, particularly as initial treatment for RRMS, could deliver optimal health outcome to patients. Benefits include improved patient quality of life (29), reduced disability (30, 31), and delayed disease progression (32), contributing not only to the individual wellbeing of MS patients but also to reduced healthcare burdens through fewer hospitalisations and interventions (33, 34). These patterns help contextualise the increased use of high-efficacy DMTs among patients and the healthcare system, despite their higher acquisition costs.

Furthermore, this study also provides insights to inform future cost-effectiveness evaluation of new drugs. Notable factors include uptake rates of new treatments after listing, time on treatment, and patient preferences. Aside from clinical differences, other considerations such as treatment convenience were noted to shape the choice and use of therapies. Observed trends indicated an increasing interest in long-acting treatments, likely associated with dosing convenience. These factors may warrant greater emphasis on HTA evaluations of new drugs, particularly for treatments of chronic diseases or disorders.

Overall, this study harnessed the strength of robust real-world administrative data with detailed drug dispensing information at individual patient level, spanning an extensive 11-year period, to provide a comprehensive view of DMT usage patterns in Australia. We focused on leveraging the information available from this dataset and prudently picked and developed indicators for evaluating rational drug use and resource allocation. Key strengths of our approach include detailed insights drawn directly from real-world data, including patient uptake, switching behaviours, treatment durations, and direct per-patient drug costs. These insights are especially valuable for healthcare stakeholders and payers in effectively managing drug usage following reimbursements and informing future budget impact analyses for new therapies. The methodology used in this study offers high scalability and generalisability for supporting broader postmarket drug monitoring, detecting of irrational use of drugs, and enhancing budget impact analysis and control. The findings can also provide valuable evidence to inform decision-making regarding listing new drugs or delisting existing drugs in the reimbursement list. For example, the findings of this study revealed usage patterns that vindicated the PBS decisions of delisting specific RRMS treatments, thereby validating the rationale behind such adjustments.

## 5 Limitation and future study

This study is descriptive in nature and based on PBS dispensing data. It does not capture clinical outcomes, disease severity, or non-drug healthcare costs, and therefore cannot directly assess comparative effectiveness, prescribing appropriateness, or cost-effectiveness. The findings should be interpreted as evidence to inform postmarket monitoring and policy. Despite the use of indication-specific PBS item codes, some misclassification was possible, and a very small number of patients with progressive forms of MS may have been included. This limitation is inherent to claims-based analyses.

In addition, per-patient drug costs were estimated using published DPMQ prices, which are indicative only. Actual net prices paid by the government are subject to confidential pricing and risk-sharing arrangements, and were not available for this study. As a result, the estimated costs may not fully reflect true government expenditure.

Future research incorporating linked clinical outcomes, safety data, and confidential pricing information would enable more robust assessment of the real-world economic and clinical implications of DMT use.

## 6 Conclusion

This study used both national and patient-level data to examine treatment patterns in RRMS in Australia following the introduction of new therapies. Our findings provide important insights into the rational use of medications and associated healthcare resources. The increase in PBS expenditure on DMTs aligned with the rising prevalence of MS during the same period. While this prevalence-driven demand was the primary factor, additional contributors included shifts in the treatment landscape from LE to HE therapies and the longer durations of treatment across different groups. Moreover, an analysis of individual DMT usage patterns revealed notably high dosages of interferon beta-1a for RRMS, resulting in increased treatment costs. These findings highlight both evolving treatment trends and areas for optimising resource allocation to manage expenditures effectively.

The findings of this study also aligned with the PBS reviews and decision to delist interferon beta-1a, reinforcing the suitability of our approach and methodology for leveraging claims data to assess the postmarket rational use of medicines. This provides actionable evidence to support resource optimisation and ensure sustainable healthcare practices.

## Data Availability

The data analysed in this study are subject to the following licenses/restrictions: The PBS dispensing data used in this study are managed by Services Australia and are not publicly available. The authors are not permitted to share these data directly. Access may be granted to eligible researchers through application to Services Australia in accordance with relevant data governance and ethics approval requirements. Requests to access these datasets should be directed to https://www.servicesaustralia.gov.au/statistical-information-and-data?context=22.
